# Neuromotor and Cognitive Outcomes of Preterm Infants at 3 Months of Corrected Age in a Northeastern Region of Brazil: Longitudinal Feasibility Study

**DOI:** 10.2196/85381

**Published:** 2026-05-22

**Authors:** Nathália de Figueiredo Silva, Carolina Daniel de Lima-Alvarez, Stacey Dusing, Silvana Alves Pereira

**Affiliations:** 1 Universidade Federal do Rio Grande do Norte Natal, Rio Grande do Norte Brazil; 2 University of Southern California Los Angeles, CA United States

**Keywords:** prematurity, motor development, cognitive development, early intervention, physical therapy

## Abstract

**Background:**

Preterm infants (PTIs) are at increased risk of neurodevelopmental impairment, particularly in socioeconomically vulnerable regions, where access to early intervention services is limited. However, little is known about the feasibility of longitudinal developmental monitoring in such contexts.

**Objective:**

This study aimed to evaluate the feasibility of recruiting, retaining, and assessing preterm infants in a resource-limited setting in Northeastern Brazil and to generate preliminary data on early neurodevelopmental outcomes.

**Methods:**

This longitudinal feasibility study was conducted at a referral maternity hospital in Northeastern Brazil between October 2023 and March 2024. PTIs born at ≤32 weeks of gestational age were recruited during hospitalization. Feasibility outcomes included recruitment rate, retention rate, and completeness of assessments. Development was assessed at three time points: (1) Neonatal Intensive Care Unit discharge, (2) Kangaroo Neonatal Intermediate Care Unit discharge, and (3) at 3 months of corrected age (CA), using the Motor Optimality Score (MOS), Test of Infant Motor Performance (TIMP), and Bayley Scales of Infant Development III (BAYLEY-III). Caregiver-infant interaction was assessed using the Recorded Interaction Task (RIT).

**Results:**

Of 45 eligible infants, 20 (44.4%) were enrolled, and 10 (50%) completed follow-up in 3 months CA. A total of 67 assessments were conducted. Attrition was due to death (n=5), loss of contact (n=2), withdrawal (n=2), and transfer to another service (n=1). Among infants assessed at 3 months CA, 60% (n=6) of infants had MOS <25, indicating increased risk of neurological impairment. At TIMP, 80% (n=8) showed motor performance below expected levels in 3 months. BAYLEY-III assessments (n=8) indicated below-average motor performance in 50% (n=4) of the infants and below-average cognitive performance in 50% (n=4). All caregiver-infant dyads assessed at follow-up (n=10) demonstrated adequate interaction on RIT (mean score 57.9, SD 12.3).

**Conclusions:**

Longitudinal developmental monitoring of PTIs in a resource-limited setting was feasible, although retention remained a major challenge. Preliminary findings indicated frequent motor and cognitive vulnerabilities after hospital discharge, underscoring the importance of structured follow-ups in early infancy. These findings support the need for larger studies to refine follow-up strategies and evaluate early intervention in similar contexts.

## Introduction

The World Health Organization defines preterm infants (PTIs) as those born before 37 weeks of gestation [1]. Preterm birth is associated with an increased risk of adverse neurodevelopmental outcomes, including impairments in motor, cognitive, language, and behavioral domains that may persist into adolescence and adulthood, affecting academic performance, mental health, and functional outcomes [2-5]. Early identification of developmental delays and timely intervention are essential to mitigate these risks and promote optimal developmental trajectories.

Early intervention programs, including sensorimotor stimulation and family-centered care, have been shown to improve outcomes in PTIs, particularly when initiated during critical periods of brain development [6,7]. However, in low- and middle-income settings, access to such services remains limited. In Brazil, especially in the northeastern region, structural challenges such as limited health care resources, shortage of trained professionals, and poor integration of care networks often result in delayed or absent developmental follow-up [8-10]. Previous studies conducted in this context have highlighted important gaps in care. Analyses of health system data have shown that a small proportion of PTIs receive early stimulation services during the first year of life (previous unpublished analyses conducted by our group; Multimedia Appendix 1). Additionally, low adherence to postdischarge follow-up (previous unpublished analyses conducted by our group; Multimedia Appendix 1) and limited family understanding of prematurity and neurodevelopment have been reported [11], suggesting barriers to continuity of care and early intervention. Together, these findings indicate a potential gap between hospital discharge and access to developmental monitoring and support.

Despite the recognized importance of early monitoring, there is limited evidence on the feasibility of conducting longitudinal developmental assessments of PTIs in resource-limited settings. Understanding the practical challenges of recruitment, retention, and assessment implementation is essential for designing larger studies and effective intervention programs. Therefore, this study aimed to evaluate the feasibility of recruiting, retention, and longitudinal developmental assessment of PTIs from hospitalization to 3 months of corrected age (CA) in a resource-limited setting in Northeastern Brazil as well as to generate preliminary data on early neurodevelopmental outcomes.

## Methods

### Study Design, Settings, and Study Procedures

This was a longitudinal feasibility study conducted at a referral maternity hospital for high-risk pregnancies in Northeastern Brazil between October 2023 and March 2024. The maternity hospital follows the Kangaroo Method [12], which is structured into 3 stages. The first stage begins during the prenatal period, with follow-up of high-risk pregnancies, and extends into the neonatal intensive care unit (NICU), where parents are encouraged to adopt skin-to-skin contact as soon as clinically possible. In this stage, physical therapy primarily focuses on cardiorespiratory and sensory systems, with sessions offered 1-2 times per day for 7 days a week. The second stage occurs in the Kangaroo Neonatal Intermediate Care Unit (KaNICU), when the neonate is clinically stable and no longer requires ventilatory support. During this phase, the mother-infant dyad is admitted together, with intensive promotion of skin-to-skin contact and direct breastfeeding. In addition, physical therapy interventions expand to include neuromotor regulation techniques while continuing sensory care, with sessions offered at least once per day for 5 days a week. The third stage occurs after hospital discharge and consists of outpatient follow-up until the infant reaches a weight of 2500 grams, after which the infant is referred to a primary health care unit near their home municipality. In this third stage, outpatient physical therapy services are available 2 days per week, with infants typically scheduled for at least 1 session per month. However, in this setting, structured developmental monitoring after hospital discharge is not routinely implemented. Study-specific procedures were implemented to provide structured longitudinal monitoring during the early postdischarge period.

### Participants and Sampling

Participants were selected using a convenience sampling approach. Eligible participants were PTIs born at ≤32 weeks of gestational age, with the absence of congenital malformation in the musculoskeletal system and genetic syndromes, and admitted to the study site during the recruitment period. Recruitment occurred over 6 months. Mothers were invited to participate during the first week of the infant’s life while the infant was hospitalized in the NICU. The invitation included an explanation of the study, followed by the importance of monitoring development for premature infants. The refusals to the invitation were respected, and no specific reason for nonparticipation was declared. All eligible infants admitted during the study period were consecutively invited to participate. As this was a feasibility study, no formal sample size calculation was performed. The sample size was considered sufficient to assess feasibility outcomes such as recruitment, retention, and completeness of assessments.

### Feasibility Outcomes

The primary objective of this study was to assess feasibility. The following feasibility outcomes were evaluated:

1. Recruitment rate: the proportion of eligible infants who were enrolled in the study.
2. Retention rate: the proportion of enrolled participants who completed follow-up at 3 months CA.
3. Attrition: reasons for loss to follow-up, including death, withdrawal, loss of contact, and transfer.
4. Completeness of assessments: the proportion of planned assessments completed at each time point.
5. Acceptability of procedures: indirectly inferred from caregiver participation and adherence to scheduled assessments.

These outcomes were used to evaluate the feasibility of conducting longitudinal developmental monitoring in this context and to inform future studies.

### Preliminary Developmental Outcomes

The secondary objective of this study was to assess preliminary developmental outcomes, specifically motor and cognitive development and parent-child interaction.

### Developmental Assessment Instruments

#### Motor Optimality Score

The Motor Optimality Score (MOS), based on Prechtl’s General Movements Assessment, was used to evaluate spontaneous motor behavior from birth to approximately 20 weeks post term. It has demonstrated validity for the early identification of infants at risk for neuromotor impairment. Scores below established cutoffs indicate increased neurological risk [13,14].

#### Test of Infant Motor Performance

The Test of Infant Motor Performance (TIMP) is a validated instrument used to assess motor performance in infants from 34 weeks of gestation to 4 months CA. It has strong psychometric properties, including high intrarater and interrater reliability and is widely used in both clinical and research settings [15].

#### Bayley Scales of Infant and Toddler Development, Third Edition

The Bayley Scales of Infant and Toddler Development, Third Edition (BAYLEY-III) is considered a gold standard instrument for assessing infant development and has been validated for use in the Brazilian population [16]. The cognitive and motor subscales were administered at 3 months CA.

### Caregiver-Infant Interaction Assessment: Recorded Interaction Task

The Recorded Interaction Task (RIT) was used to assess caregiver-infant interaction during a routine diaper-changing activity at 3 months CA. This tool has demonstrated validity for evaluating bonding and interaction quality. Scores below 35 indicate potential interaction difficulties [17].

**Figure 1:**
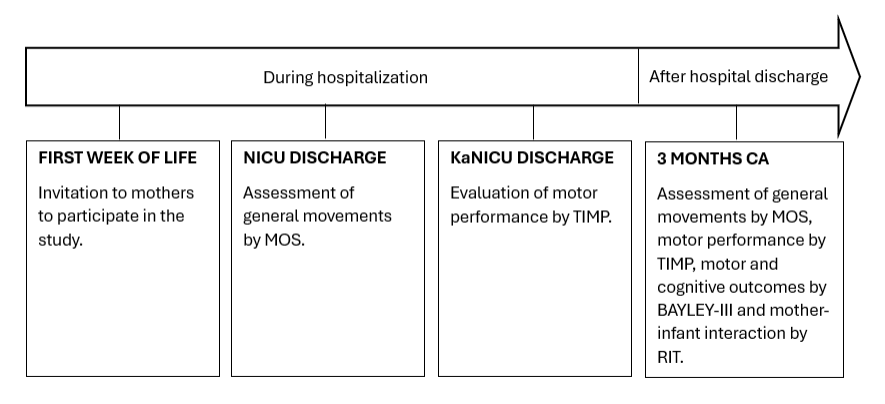
Timeline of developmental assessments performed in preterm infants from hospitalization to 3 months of corrected age in a longitudinal feasibility study conducted at a referral maternity hospital in Northeastern Brazil. BAYLEY-III: Bayley Scales of Infant and Toddler Development Third Edition; CA: corrected age; KaNICU: Kangaroo Neonatal Intermediate Care Unit; MOS: Motor Optimality Score; NICU: neonatal intensive care unit; RIT: recorded interaction task; TIMP: Test of Infant Motor Performance.

Figure 1 presents a timeline outlining the instruments and corresponding assessment points conducted at a referral maternity hospital in Northeastern Brazil between October 2023 and March 2024. These instruments were selected due to their established validity, reliability, and sensitivity for detecting early developmental changes in PTIs. The MOS and TIMP, although requiring specific training for proper administration and interpretation, are relatively low-cost and accessible tools, making them suitable for use in resource-limited settings. The RIT is a simple, low-cost instrument that allows the assessment of caregiver-infant interaction through brief video-recorded routines, and its cross-cultural adaptation to the Brazilian context is currently being conducted by our research group. In contrast, the BAYLEY-III, considered the gold standard for developmental assessment, was included to provide greater robustness and comprehensiveness to the developmental data. However, the BAYLEY-III requires specialized training and higher costs, which may limit its routine use in resource-limited settings.

### Data Collection Procedures

Infants were followed longitudinally from hospitalization until 3 months CA. Developmental assessments were conducted at three time points: (1) at NICU discharge, (2) at KaNICU discharge, and (3) at 3 months of CA.

All assessments were conducted by a trained physical therapist. Postdischarge assessments were conducted at the maternity hospital’s outpatient clinic. Appointments were scheduled prior to discharge and reinforced through biweekly phone contact. All evaluations were video recorded and analyzed offline by trained professionals. Video recordings were obtained during in-person assessments by the trained evaluator and were part of the study-specific procedures. Approximately 75% (50) of the assessments were independently coded by a second evaluator blinded to the infant’s clinical condition.

### Statistical Analysis

Data were analyzed using the SPSS software (version 20.0; IBM Corp). Descriptive statistics were used to summarize the data. Continuous variables were expressed as median and interquartile range, while categorical variables were presented as frequencies and percentages. Given the exploratory nature of this feasibility study and the small sample size, no inferential statistical analyses were performed.

### Ethical Considerations

This study was approved by the Research Ethics Committee of the Federal University of Rio Grande do Norte (CAEE protocol: 60615722.0.0000.5537) and complied with the requirements for research involving human subjects, as outlined in resolution number 466 of the Brazilian National Research Ethics Commission (CONEP). Written informed consent was obtained from all participants’ caregivers before enrollment. All data were handled in accordance with ethical standards to ensure confidentiality and privacy. Participant data were deidentified before analysis. No financial compensation was provided to participants, in accordance with Brazilian ethical regulations for research involving human subjects (resolution 466/2012), which prohibit payment for participation.

## Results

### Participant Flow and Feasibility Outcomes

**Figure 2:**
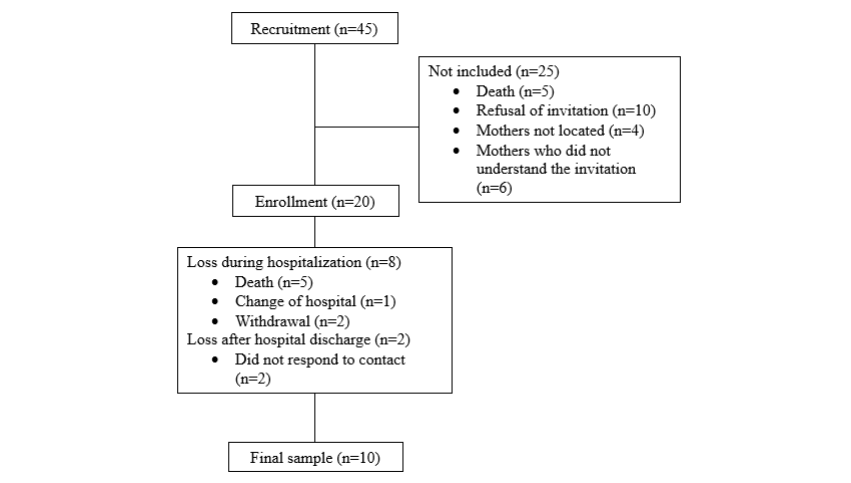
Flow diagram of participant recruitment, enrollment, and final sample in a longitudinal feasibility study of preterm infants conducted in Northeastern Brazil.

Figure 2 presents the participant flow. Table 1 presents the characteristics of the 20 PTIs included in the study.

**Table 1:** Baseline characteristics of preterm infants enrolled in a longitudinal feasibility study conducted at a referral maternity hospital in Northeastern Brazil between October 2023 and March 2024.

Characteristics		Values
**Infant characteristics, median (IQR)**		
	Gestational age at birth (weeks)	30.05 (2.8)
	Birth weight (grams)	1215 (480)
	Apgar score^a^ at 1 min	7 (4)
	Apgar score at 5 min	8 (1.25)
**Sex (n=20), n (%)**		
	Female	11 (55)
	Male	9 (45)
**Physical therapy after discharge (n=10), n (%)**		
	Yes	0 (0)
	No	10 (100)

^a^Apgar: neonatal vitality score, with scores below 4 indicating low neonatal vitality.

### Feasibility Outcomes

Overall, the feasibility outcomes indicate moderate success in participant recruitment and high acceptability of study procedures among retained participants but substantial challenges in retention and completeness of assessments over time. The attrition observed after hospital discharge and the occurrence of incomplete assessments due to infant illness highlight important barriers to longitudinal follow-up in this setting. These findings underscore the need to consider both clinical vulnerability and contextual factors when designing future studies involving PTIs in similar resource-limited environments. Table 2 shows the feasibility outcomes.

**Table 2:** Feasibility outcomes of a longitudinal study assessing developmental monitoring in preterm infants from hospitalization to 3 months of corrected age in Northeastern Brazil.

Developmental monitoring	Feasibility outcome
Recruitment rate (n=45), n (%)	20 (44.4)
Retention rate (n=20), n (%)	10 (50)
Attrition rate	Death (n=5); withdrawal (n=2); transfer (n=1); loss of contact (n=2)
Completeness of assessments	Completeness of assessments was 80% at 3 months of corrected age, as 8 out of 10 participants completed all planned evaluations. Two participants had incomplete data due to intercurrent illness, which prevented the administration of the scheduled assessments
Acceptability of procedures	Acceptability of study procedures was high, as all retained caregiver-infant dyads (n=10) attended the scheduled follow-up visit and agreed to participate in all proposed assessments, including video-recorded procedures

A total of 67 assessments were conducted across all time points, including 21 MOS, 22 TIMP, 8 BAYLEY-III cognitive assessments, 8 BAYLEY-III motor assessments, and 10 RIT assessments. The completeness of assessments varied by time point, with higher completion rates during hospitalization and reduced completion at 3 months CA due to losses to follow-up.

### Developmental Outcomes During Hospitalization (n=12)

At NICU discharge, MOS ranged from 9 to 34, with a mean of 24.45 (SD 7.6). Approximately 45.5% (n=5) of the infants scored below 25, indicating increased risk for neuromotor impairment. One PTI did not undergo MOS assessment at NICU discharge due to sedative medication use. At KaNICU discharge, all infants assessed with the TIMP demonstrated motor performance within the average range for their CA. The two participants lost to follow-up due to loss of contact after hospital discharge shared similar baseline clinical characteristics. Both were classified as very preterm and presented Apgar scores greater than 8 at the fifth minute. At KaNICU discharge, both infants demonstrated average performance on the TIMP. However, their MOS assessed at NICU discharge differed, with scores of 31 and 8, respectively.

### Developmental Outcomes at 3 Months CA (n=10)

At 3 months CA, MOS ranged from 10 to 28, with a mean of 21.9 (SD 6.2). A total of 60% (n=6) of the infants scored below 25, indicating increased risk of neurological impairment. In the TIMP assessment, 80% (n=8) of the infants showed a decline in motor performance compared to hospital discharge, with only 20% (n=2) remaining in the average range. The remaining infants were classified as low average (n=4, 40%), below average (n=3, 30%), or well below average (n=1, 10%).

BAYLEY-III assessments were completed in 8 infants. For motor development, 50% (n=4) were classified as average and 50% (n=4) as below average (low average). For cognitive development, 50% (n=4) were classified as average, while 50% (n=4) were below average (n=2, 25% low average and n=2, 25% borderline).

### Caregiver-Infant Interaction (n=10)

All caregiver-infant dyads assessed using the RIT demonstrated adequate interaction, with a mean score of 57.9 (SD 12.3; range 38-76), above the cutoff indicating concern.

### Individual Developmental Trajectories

Individual developmental trajectories for the 8 infants who completed all assessments are presented in Table 3. Notably, several infants who initially presented with favorable scores during hospitalization exhibited subsequent declines in motor performance and increased neurological risk indicators at follow-up. The exclusion of two participants due to illness highlights the clinical vulnerability of this population and its impact on data completeness.

**Table 3:** Individual developmental trajectories of preterm infants in a longitudinal feasibility study conducted in Northeastern Brazil.

	During hospitalization		At 3 months of corrected age			
ID	MOS^a^	TIMP^b^	TIMP	MOS	BAYLEYm^c^	BAYLEYc^d^
1	20#^e^	Average	Low-average	21#	Average	Average
2	34	Average	Far below average	26	Average	Low average
3	31	Average	Below average	10#	Low average	Borderline
4	18#	Average	Average	25	Average	Average
5	*^f^	Average	Low-average	28	Low average	Borderline
6	26	Average	Below average	12#	Low average	Average
7	23#	Average	Low-average	28	Low average	Average
8	19#	Average	Below average	22#	Average	Low average
9	25	Average	Below average	23#	Ø^g^	Ø
10	33	Average	Average	24#	Ø	Ø

^a^MOS: Motor Optimality Score.

^b^TIMP: Test of Infant Motor Performance.

^c^BAYLEYm: Bayley Scales of Infant and Toddler Development III – motor subscale.

^d^BAYLEYc: Bayley Scales of Infant and Toddler Development III – cognitive subscale.

^e^#: score suggestive of neurological deficit and need for intervention.

^f^*: not performed due to use of sedative medication.

^g^Ø: assessment not performed due to intercurrent illness.

## Discussion

This feasibility study evaluated the recruitment, retention, and implementation of longitudinal developmental assessments in PTIs in a resource-limited setting in Northeastern Brazil. The findings demonstrated moderate recruitment rates, high acceptability of study procedures among retained participants, and substantial challenges in retention and completeness of assessments over time. Additionally, preliminary developmental results suggested a decline in motor performance after hospital discharge and a high proportion of infants at risk for neurodevelopmental impairment.

The moderate recruitment rate observed in this study is consistent with previous feasibility studies involving PTIs in similar contexts. Recruitment in neonatal populations is often influenced by parental stress, clinical instability of infants, and logistical constraints during hospitalization [18]. Additionally, in this study, participant invitation occurred within the first week of the infant’s life, a period characterized by heightened emotional vulnerability among mothers following preterm birth. This early postpartum phase is frequently associated with increased levels of stress, anxiety, and psychological distress, which may negatively impact decision-making and willingness to participate in research [19].

However, the most critical feasibility challenge identified in this study was the high attrition rate after hospital discharge, which reflects a well-documented barrier in longitudinal follow-up programs for PTIs [20]. In this study, 40% of the follow-up losses were due to participant withdrawal and loss of contact. This may be partially explained by the geographical characteristics of the study setting, as the referral maternity hospital serves a large territorial area of approximately 3565 km² across 15 municipalities. Greater distance between participants’ residences and health care facilities has been consistently associated with reduced adherence to follow-up and continuity of care, particularly in vulnerable populations, due to transportation difficulties, costs, and access barriers [21]. In addition, recent evidence highlights that maintaining participation in follow-up programs is particularly challenging in low- and middle-income countries due to socioeconomic factors, transportation barriers, and limited integration of post-discharge care services [22]. A recent feasibility study conducted in Brazil using a hybrid (telehealth and in-person) follow-up model reported higher retention rates and improved adherence, suggesting that alternative follow-up strategies may mitigate these challenges [23]. Similarly, digital and web-based follow-up tools have been proposed as feasible solutions to improve continuity of care and parental engagement in early developmental monitoring [24]. The use of such health technologies may represent a promising and effective alternative for monitoring PTIs in this context, particularly by reducing geographic and logistical barriers to follow-up. In Brazil, data from the TIC Domicílios 2024 report indicate that a substantial proportion of the population, including those in more socioeconomically vulnerable regions, access the internet primarily through mobile phones, which may facilitate the implementation of digital health strategies for follow-up and monitoring [25].

In contrast to retention, acceptability of the study procedures was high among participants who remained in the study, as all caregiver-infant dyads attended follow-up visits and agreed to participate in the assessments. This finding aligns with previous research demonstrating high parental satisfaction and engagement in structured developmental follow-up, including remote or video-based assessments [26]. These results suggest that, once barriers to access are overcome, caregivers are willing to engage in early developmental monitoring. Additionally, a study conducted in the same region reported that caregivers of PTIs have a limited understanding of prematurity and its associated neurodevelopmental outcomes [11]. This suggests that investing in strategies to improve caregiver knowledge, particularly during hospitalization, may further enhance acceptability and engagement in developmental monitoring programs.

The reduced completeness of assessments observed in this study, partially due to infant illness, highlights the clinical vulnerability of this population. PTIs are at increased risk of medical complications and rehospitalization, which can interfere with scheduled follow-up and data collection [5]. This challenge has also been reported in longitudinal studies of PTI, where health instability contributes to missing data and follow-up discontinuity [20].

Regarding developmental outcomes, the observed decline in motor performance between hospital discharge and 3 months of CA is clinically relevant. Early developmental care and continuous monitoring are essential to identify and address emerging delays. In Brazil, referral to early intervention and rehabilitation services for high-risk infants often occurs after the recommended screening window for early neurodevelopmental milestones (4 months of CA) due to systemic barriers in the integration of care networks, creating a gap in stimulation during the critical period of rapid neurological development [27-29]. Recent reviews emphasize that structured follow-up programs with early detection and intervention can significantly improve neurodevelopmental outcomes in PTIs [7]. The absence of postdischarge rehabilitation in this study population may have contributed to the observed decline, reinforcing the importance of continuity of care after hospital discharge.

Taken together, these findings suggest that the main barriers to feasibility in this context are not related to the acceptability of the procedures themselves but rather to external and contextual factors affecting follow-up continuity. This distinction is critical for the design of future studies and interventions. This study has several limitations that should be considered. First, the small sample size and the reduced number of participants completing follow-up (n=10, with only 8 completing all assessments) limit the generalizability of the findings and preclude inferential statistical analysis. Second, the high attrition rate may have introduced selection bias, as participants who completed follow-up may differ systematically from those lost to follow-up. Third, the study was conducted in a single center, which may limit the applicability of the findings to other settings. Although telehealth strategies were identified as promising approaches to improve retention, they were not implemented in this study due to resource and infrastructure limitations at the time of data collection. Future studies should consider incorporating hybrid or remote monitoring strategies to enhance feasibility.

This study demonstrates that the longitudinal developmental monitoring of PTIs in a resource-limited setting is feasible, with moderate recruitment and high acceptability of procedures. However, significant challenges related to retention and completeness of assessments were identified, particularly after hospital discharge. These findings highlight the need for strategies to improve follow-up continuity, such as alternative care models and strengthened postdischarge support. Future larger-scale studies are warranted to evaluate the effectiveness of such strategies and to further investigate early developmental outcomes in this population.

## Appendix

Multimedia Appendix 1 Summary of prior analyses conducted by the research group on follow-up adherence in preterm infants within the Kangaroo Method context, and epidemiological analysis on access to early stimulation among preterm infants in Brazil.

## Abbreviations

BAYLEY-III: Bayley Scales of Infant and Toddler Development, Third Edition
CA: corrected age
KaNICU: Kangaroo Neonatal Intermediate Care Unit
MOS: Motor Optimality Score
NICU: neonatal intensive care unit
PTI: preterm infant
TIMP: Test of Infant Motor Performance

## Data Availability

The datasets used and/or analyzed during this study are available from the corresponding author on reasonable request.
