# Supplementary material for: Neuromotor and Cognitive Outcomes of Preterm Infants at 3 Months of Corrected Age in a Northeastern Region of Brazil: Longitudinal Feasibility Study
Source: JMIR Form Res. 2026 May 22;10:e85381. doi: 10.2196/85381 (PMC13241792; doi:10.2196/85381)
Supplement: Multimedia Appendix 1 [file formative_v10i1e85381_app1.docx]

**Appendix 1. Summary of prior analyses conducted by the research group on follow-up adherence in preterm infants within the Kangaroo Method context**

This appendix summarizes findings from an ecological observational study conducted by our research group in a public maternity hospital in northeastern Brazil, aiming to investigate factors associated with adherence to outpatient follow-up in preterm infants.

Study objective

To evaluate whether maternal satisfaction with participation in neonatal care during hospitalization is associated with attendance at outpatient physical therapy follow-up in the third stage of the Kangaroo Method.

Methods

The study included 30 mothers of preterm infants (<37 weeks gestational age) recruited from a tertiary public maternity hospital. Maternal satisfaction was assessed using the Brazilian version of the EMPATHIC-N questionnaire, focusing on the domain of parental participation. Follow-up attendance data were collected for the first month after hospital discharge. Descriptive and inferential analyses were conducted, including Wilcoxon and Spearman correlation tests.

Key findings

- Maternal satisfaction with participation in neonatal care was high, particularly in the Kangaroo Intermediate Neonatal Care Unit compared to the NICU (p = 0.003).
- Despite high satisfaction levels, adherence to outpatient physical therapy follow-up was extremely low, with only 8% of families attending scheduled visits.
- A significant negative correlation was observed between maternal satisfaction during NICU hospitalization and physical therapy follow-up attendance.
- A high proportion of infants (57%) presented neuroimaging findings associated with increased risk of developmental delay, indicating a mismatch between clinical need and follow-up adherence.

Interpretation

These findings suggest that maternal satisfaction with participation in inpatient care does not necessarily translate into engagement with post-discharge developmental follow-up. Structural, educational, and systemic barriers may play a more significant role in determining continuity of care.

Relevance to the present study

This prior analysis informed the rationale for the present feasibility study by highlighting critical gaps in follow-up adherence and suggesting that additional strategies are needed to improve engagement in early developmental monitoring programs for preterm infants.

**Appendix 2. Epidemiological analysis on access to early stimulation among preterm infants in Brazil**

This appendix summarizes findings from an ecological study conducted by our research group examining access to early stimulation procedures for preterm infants within the Brazilian public health system.

Study objective

To analyze the ratio between the number of early stimulation procedures and preterm births performed by the Brazilian Unified Health System (SUS) between 2018 and 2022.

Methods

This retrospective ecological study used publicly available data from DATASUS. Data on preterm live births were obtained from the Live Birth Information System (SINASC), and records of early stimulation procedures for neuropsychomotor development were collected from the Outpatient Information System (SIA/SUS). Analyses were conducted at national, regional, and state levels. The ratio between early stimulation procedures and preterm births was calculated, and exploratory spatial analysis was performed using thematic maps.

Key findings

- At the national level, the ratio of early stimulation procedures to preterm births was 0.13.
- This ratio indicates insufficient coverage for even a single early stimulation consultation per preterm infant during the analyzed period.
- All regions of the country showed similarly insufficient ratios.
- Marked regional and state-level disparities in access to early stimulation services were observed.

Interpretation

These findings reveal a substantial mismatch between the number of preterm live births and the provision of early stimulation procedures within the public health system, indicating limited access to early developmental follow-up during a critical period.

Relevance to the present study

This analysis provides important contextual evidence supporting the need to evaluate the feasibility of structured follow-up strategies, particularly in settings where access to early developmental monitoring and intervention services is limited and unevenly distributed.
